# Supplementary material for: Effects of Social Cues on Biosecurity Compliance in Livestock Facilities: Evidence From Experimental Simulations
Source: Front Vet Sci. 2020 Mar 27;7:130. doi: 10.3389/fvets.2020.00130 (PMC7120031; doi:10.3389/fvets.2020.00130)
Supplement: Supplementary file 1 [file Data_Sheet_1.pdf]

Supplementary Materials

2.4.2 Extended: Ratio of variances

In the ratio of variances analysis we control for the effect of the two main variables, infection risk and message delivery method. Implicit in this analysis is the assumption that the psychological distance effect is uniform across the three distributions of paired social cue treatments. Here we first present the mean of the psychological distance effect for the three paired social cue treatments.

**Paired Social Cue Treatments**	**Mean Psychological Distance Effect**
SC1 vs. SC2	0.005
SC1 vs. Control	-0.013
SC2 vs. Control	-0.017

Table 1: Mean psychological distance effect for paired social cue treatments used in ratio of variances analysis. SC1 corresponds with Compliance by Coworker treatments, SC2 corresponds with Non-Compliance by Coworker treatments, Control corresponds with Coworker Control treatments.

The uniform distribution of the psychological distance effect here is due to stochasticity in treatment order and infection events. Here we now present the results of three t-tests which compare the mean psychological distance effect between the paired social cue treatments. None of the distributions were found to have a significantly different mean. Based on this result the psychological distance effect was not included as a factor in the ratio of variances analysis.

		***P*-value**	
SC1 vs. SC2	0.005	0.488	0.366
SC1 vs. Control	-0.013	1	0.828
	**Mean Psychological Distance Effect**	-0.013	-0.017
		SC1 vs. Control	SC2 vs. Control

Table 2: Results of t-test to compare mean psychological distance effects for paired social cue treatments. SC1 corresponds with Compliance by Coworker treatments, SC2 corresponds with Non-Compliance by Coworker treatments, Control corresponds with Coworker Control treatments.

Introductory Slideshow

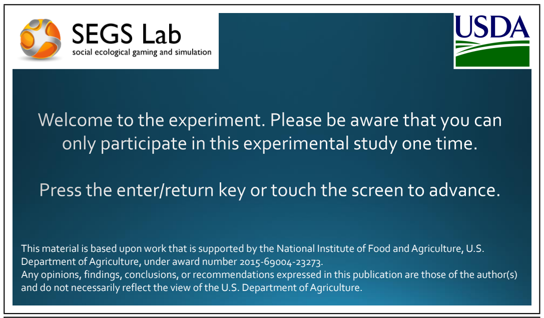

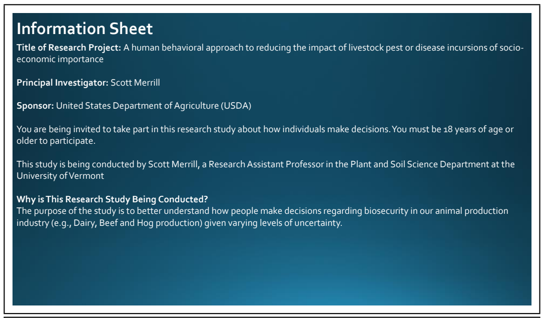

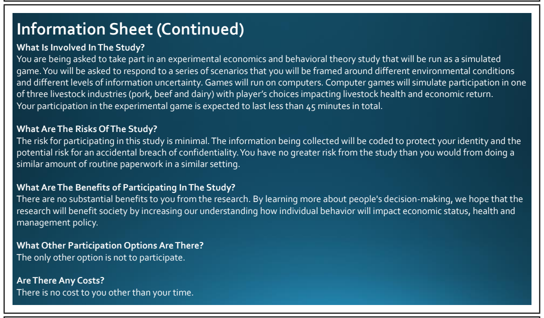

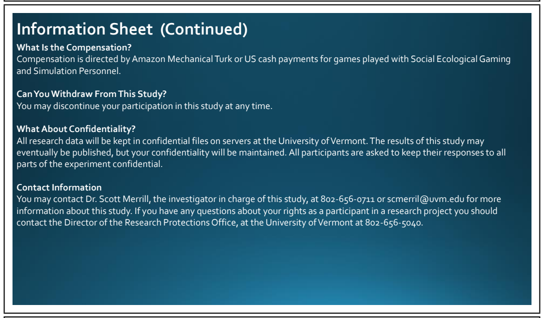

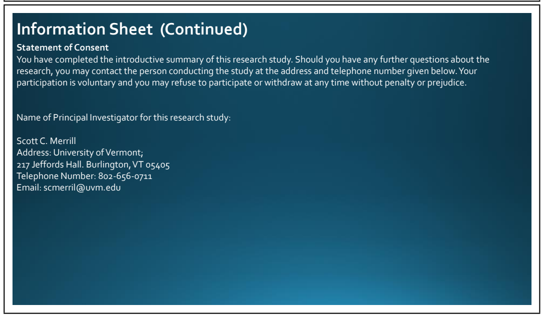

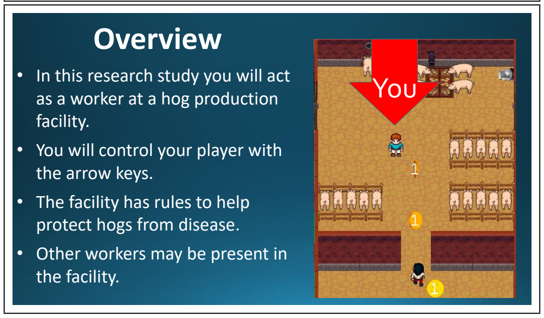

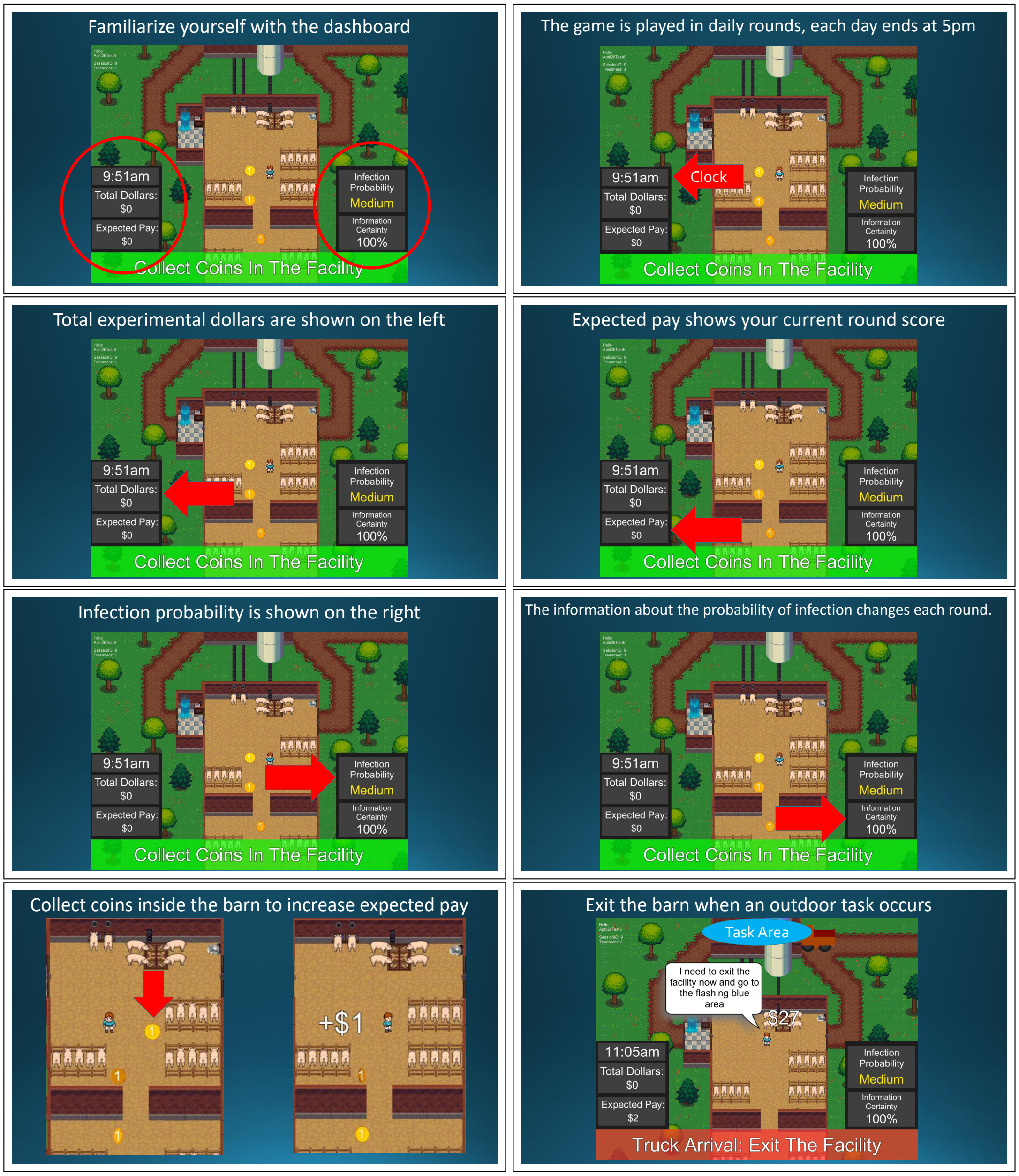
Familiarize yourself with the dashboard
9:51am
Total Dollars:
$0
Expected Pay:
$0
Infection Probability
Medium
Information Certainty
100%
Collect Coins In The Facility
The game is played in daily rounds, each day ends at 5pm
Clock
Total experimental dollars are shown on the left
Expected pay shows your current round score
Infection probability is shown on the right
The information about the probability of infection changes each round.
Collect coins inside the barn to increase expected pay
+$1
Exit the barn when an outdoor task occurs
Task Area
I need to exit the facility now and go to the flashing blue area
$27
11:05am
Expected Pay:
$2
Truck Arrival: Exit The Facility

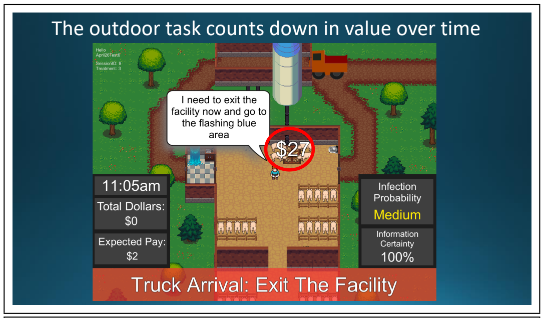
The outdoor task counts down in value over time
I need to exit the facility now and go to the flashing blue area
$27
11:05am
Total Dollars: $0
Expected Pay: $2
Infection Probability
Medium
Information Certainty
100%
Truck Arrival: Exit The Facility

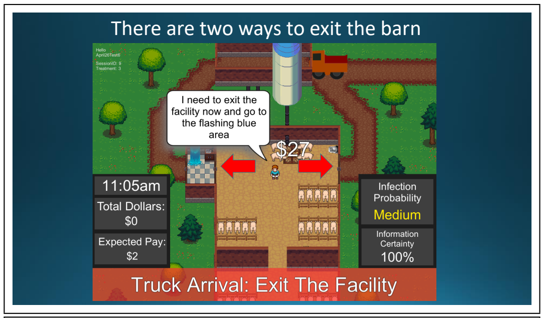
There are two ways to exit the barn
I need to exit the facility now and go to the flashing blue area
$27
11:05am
Total Dollars: $0
Expected Pay: $2
Infection Probability
Medium
Information Certainty
100%
Truck Arrival: Exit The Facility

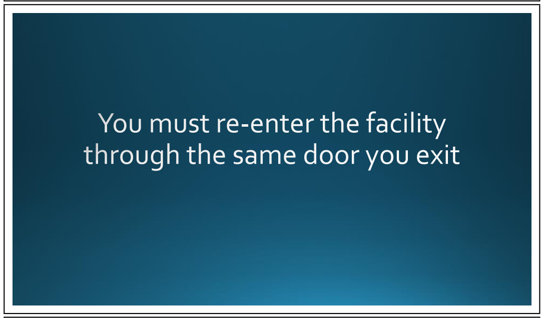
You must re-enter the facility through the same door you exit

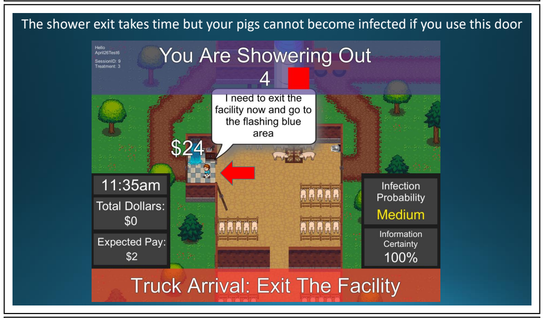
The shower exit takes time but your pigs cannot become infected if you use this door
You Are Showering Out
4
I need to exit the facility now and go to the flashing blue area
$24
11:35am
Total Dollars: $0
Expected Pay: $2
Infection Probability
Medium
Information Certainty
100%
Truck Arrival: Exit The Facility

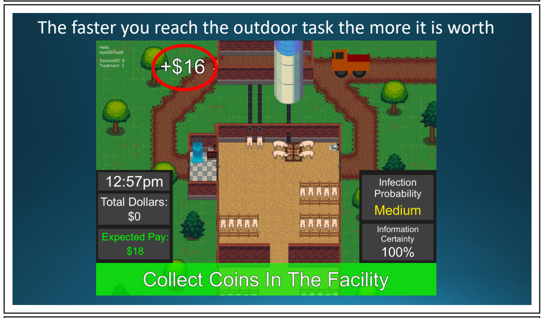
The faster you reach the outdoor task the more it is worth
+$16
12:57pm
Total Dollars: $0
Expected Pay: $18
Infection Probability
Medium
Information Certainty
100%
Collect Coins In The Facility

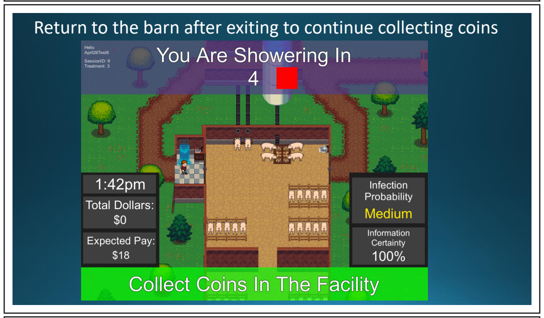
Return to the barn after exiting to continue collecting coins
You Are Showering In
4
1:42pm
Total Dollars: $0
Expected Pay: $18
Infection Probability
Medium
Information Certainty
100%
Collect Coins In The Facility

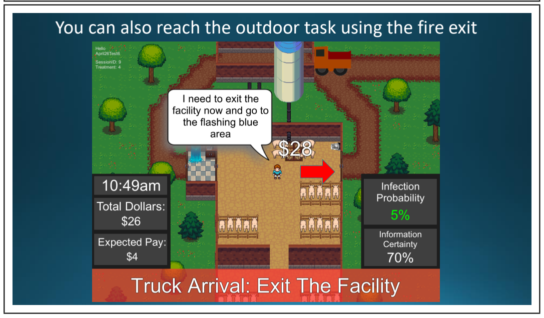
You can also reach the outdoor task using the fire exit
I need to exit the facility now and go to the flashing blue area
$28
10:49am
Total Dollars: $26
Expected Pay: $4
Infection Probability
5%
Information Certainty
70%
Truck Arrival: Exit The Facility

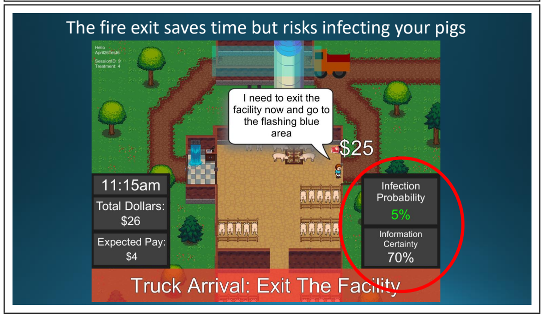
The fire exit saves time but risks infecting your pigs
I need to exit the facility now and go to the flashing blue area
$25
11:15am
Total Dollars: $26
Expected Pay: $4
Infection Probability
5%
Information Certainty
70%
Truck Arrival: Exit The Facility

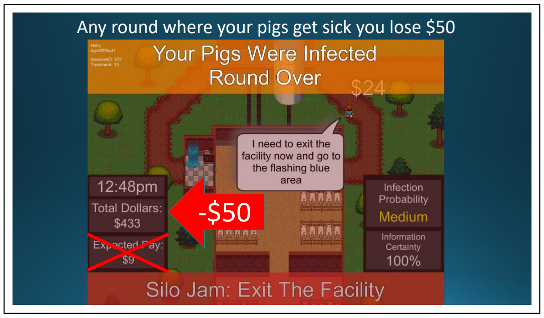

Game play

- There will be a demonstration followed by a practice round that will not count towards your total dollars
- Experimental dollars will be converted to $US at a rate of $30 to $1. For example, if you earn 30 experimental dollars you will earn $1 in United States currency
- After the all rounds are complete, you will be asked to complete a series of questions. Questions are expected to take approximately 3 minutes to answer
